# “Is a picture really worth a thousand words?”: A case study on classifying user attributes on Instagram

**DOI:** 10.1371/journal.pone.0204938

**Published:** 2018-10-05

**Authors:** Junho Song, Kyungsik Han, Dongwon Lee, Sang-Wook Kim

**Affiliations:** 1 Department of Computer Science and Engineering, Hanyang University, Seoul, Republic of Korea; 2 Department of Software and Computer Engineering, Ajou University, Suwon, Republic of Korea; 3 College of Information Sciences and Technology, Pennsylvania State University, University Park, United States of America; Universidad Nacional de Mar del Plata, ARGENTINA

## Abstract

Because using social media has become a major part of people’s daily lives, many of their personal characteristics are often implicitly or explicitly reflected in the content they share. We present a study of two personal characteristics—age and gender—related to user engagement on Instagram that can be determined through the characterization of images and tags. We demonstrate the strong influence of age and gender on Instagram use in terms of topical and content differences. We then build age and gender classification models that yield F1 scores of up to 88% and 74% in the detection of age and gender, respectively, and that better characterize users by images than by tags. We further demonstrate the robustness of our models using a new set of test data, with which the models exhibit greater overall performance than human raters. Our study highlights that future research should look to exploit images to a greater degree because they complement text and there are many unexamined images with no embedded text available.

## Introduction

Social media has penetrated daily life, allowing users to access, create, and interact with a wide range of information. Because of this strong connection between users and social media, online behaviors often reflect various user characteristics, such as age, gender, emotion, interests, attitude, and personality. These characteristics can thus often be inferred from the content that users share [1].

One common method of identifying user attributes in social media has been to use text. This is because users often share personal updates or express their thoughts in written language. Characterizing and detecting user attributes has become one of the most active research topics in natural language processing (NLP). As such, many NLP features, including linguistic style, word n-grams, and topical interests have been utilized to characterize and predict user behavior [2–12]. In addition to the use of text, user activity, which reflects how users engage and interact with others, has also been employed to determine user characteristics. For example, previous research has shown that there are differences in various activities, such as the average number of “likes” or comments received from others per image, between younger and older users [13, 14] and between different social networks [15, 16].

One approach to user characterization in social media that has been studied less thoroughly is the utilization of images. Using images, users can share content that may not be easily delivered through text, such as emotions, atmosphere. However, compared to text and user activity, images have traditionally presented a technical challenge for researchers in terms of retrieving information from them. This is less of an issue today because image recognition using deep learning techniques (e.g., convolution neural networks) can now recognize many objects within an image with reasonably high accuracy and can even extract semantic information [17]. This offers new opportunities to employ images to obtain information that is not accessible from text or user activity but that can be used to characterize users. This information can also be employed in human-computer interaction (HCI) research.

Of the NLP studies that have used text information, a majority have used blogs, which are mostly composed of complete sentences, or tweets that are 140 characters or fewer in length. A major focus has been placed on linguistic (e.g., stylistic, syntactic) features, but user-generated tags have received less attention for user characterization and prediction modeling. Although tags consist of only a single word, they can reflect image content, user intentions, and summaries of sentences, among other information, which have potential connections to user characteristics [18, 19]. Of the many online social networking sites, image-based sites such as Instagram have increased significantly in popularity and they collectively produce a large volume of content [20, 21]. One unique aspect of Instagram is that, unlike Facebook or Twitter, where text is the main content type, it operates mainly through image sharing, and hashtags rather than complete sentences are the dominant text-based activity. Images and tags have been subject to less research than text in the context of large-scale content analysis, and little is known about how users can be defined and characterized by images and tags.

In this study, we aim to investigate the following two questions: (1) How much do images and tags differ in their characterization of social media users? (2) Which types of information better predict the characteristics of social media users? In other words, does the idiom *“a picture is worth a thousand words”*, meaning that posting behavior may be better reflected through images than a text description, apply to the characterization and classification of users?

We analyze the use of tags and images in the characterization of users on Instagram and on image-based social media in general. We focus on *age* and *gender* as our target user attributes, given that they are fundamental to building a user profile. Based on data obtained from Instagram, we investigate the topical and content differences between user groups that are created based on these attributes. We then build classification models for groups of user characteristics using tags only, images only, and tags and images combined, with Term Frequency-Inverse Document Frequency [22] and word embedding (Word2Vec with bag-of-words implementation) [23, 24] as features. We investigate the differences between our machine learning models and human evaluation in classifying users to determine if tags or images provide sufficient information for user classification. Further, using human evaluation, we systematically analyze the quality and possible biases of human predictions. Our results support the following conclusions:

- **Content**
  - Topics from images and tags are distinctive enough to distinguish age and gender groups.
  - The variance in topic distribution from tags is higher than that from images.
  - Teens and females tend to have a higher ratio of selfies than adults and males.
- **Prediction**
  - Models with images outperform those with tags. Models with images and tags combined do not exhibit better performance.
  - Models with features from TF-IDF generally perform better than those with features from word embedding. Combining the features (TF-IDF + word embedding) generally increases model performance.
- **Human versus Machine Performance**
  - Our models demonstrate their robustness against new test data by comparing them with human evaluators.
  - Our models yield similar degrees of accuracy for age and gender classification.
  - Our user study highlights the challenges faced by humans in determining a user’s age and gender.

Our work contributes to a better understanding of user attributes and how those attributes are represented on social media. We demonstrate that classification models based on images outperform models using tags, as well as human evaluators. This indicates that future research should attempt to make more use of images in user classification; images act as an effective complement to text and many social media images are unaccompanied by text. Additionally, more accurate information will be able to be gathered from images as algorithms continue to evolve. Our findings can be applied to the identification of people who have similar interests or who engage in similar activities, personalized recommendations, and the detection of abnormal activities, all of which will help social media users engage with social media in a more interactive and meaningful fashion.

## Related work

### Comparative analyses of age and gender

Many studies have used variations in linguistic characteristics to identify gender differences in social media. For example, Argamon et al. [3] studied how gender was associated with the use of language in blogs. The results showed that articles and prepositions are significantly more commonly used by male bloggers, while personal pronouns, conjunctions, and auxiliary verbs are more frequent in posts by female bloggers. In terms of content-based features, male bloggers more frequently wrote about religion, politics, business, and the Internet, while female bloggers more often wrote about conversation, at-home activities, and romance. Newman et al. [9] also examined the gender differences in language use in over 14,000 text samples that were either directly written or transcribed from speech. It was found that females used more words related to psychological and social processes, while males referred more to object properties and impersonal topics. Although these effects were largely consistent across different contexts, the pattern of variation suggests that gender differences are greater for tasks that place fewer constraints on language use. Popescu and Grefenstette [11] found that female tags are more personal (e.g., roses, necklace), whereas male tags are more neutral or technical (e.g., panorama, lens).

Similarly, Thelwall et al. [25] determined that females are more likely to give and receive positive comments than males, suggesting that females are more successful social networking site users partially because of their greater ability to textually harness positive affect. This is supported by Zhang et al. [26], who investigated gender differences in hashtag use on Instagram and classified hashtags into both informative/emotional and positive/negative categories. They found that females tend to use more emotional and positive hashtags when posting images on Instagram, whereas males have a greater tendency to use informative and negative hashtags. These sentimental characteristics were also identified in a study by Ottoni et al. [15]. Their study found that females tend to describe themselves using words of affection and positive emotions, while males are more likely to describe themselves in an assertive way, using words associated with work, achievement, and money. Additionally, females tend to invest more effort in reciprocating social links and are more active and general in their content generation, whereas males tend to focus their posts on specific topics.

For social networking sites in which image activities are a heavy focus, selfies have been widely studied because users construct their identities and often express their belonging to a certain community through these. Souza et al. [27] presented a large-scale study that characterized selfies in terms of age, gender, geography, country, and other cultural variables. Their results showed that young females are the most prominent group of selfie-takers around the world. Döring et al. [28] also found that male and female Instagram users’ selfies are even more stereotypical than traditional gender stereotypes. For example, more selfies produced by females were found to fit in categories such as faceless portrayals, while male selfies often included their muscles.

Many studies have aimed to characterize age-based user groups from the perspective of use and engagement. Using an ethnographic approach, Quinn et al. [29] found that younger users (ages 15-30) tend to use different social media features (e.g., updating their status) more often than older users (ages over 50). In the same vein, Dhir et al. [30] provided empirical evidence on how adolescents (ages 12-19), young adults (ages 20-30), and adults (ages 31-50) differ in terms of selfie behavior by studying 3,763 users. They found that, compared to adults, adolescents are more likely to take individual and group selfies, post their own selfies, and use image graphic filters. They also found that young adults are more likely than older adults to take individual and group selfies, and post and edit images. Han et al. [14] divided users into teens (ages 13-19) and adults (ages 30-39) and examined their behavioral differences with respect to certain attributes such as the number of images posted, the number of likes and comments received, and the popularity of the user. Using these features, they demonstrated that the two user groups could be identified with up to 81% accuracy.

### Modeling user age and gender

Predicting and characterizing user attributes such as age and gender has become an active research topic in NLP. Several prior studies have presented machine learning-based models using NLP for text written by users. For example, Mukherjee and Liu [8] introduced a new class of features, known as variable length part-of-speech (POS) sequence patterns, which are mined from training data using a sequence pattern mining algorithm. They also proposed a new feature selection method based on an ensemble of several feature selection criteria and approaches. Based on 3,100 blogs, of which 51.2% were written by males, the highest accuracy achieved by the proposed models was 88.6%.

Burger et al. [5] used a set of different types of text, including screen names, full names, biographies, and tweets on Twitter, and extracted character n-grams and word n-grams as features. When tweet text only was used, the accuracy was 74.5% for gender prediction. The performance significantly increased to 91.8% when all features were used. Similarly, Peersman et al. [10] applied a text categorization approach for the prediction of age and gender on a chat text corpus. Their models were based on word uni-, bi-, and tri-grams, as well as character bi-, tri-, and tetra-grams. Their models achieved an accuracy of 88.8% and an F1 score of 91.7% for the classification of two age groups. Rosenthal and McKeown [12] were able to predict if a blog author was part of the pre- or post-social media generation with an accuracy of 81.5%. Al Zamal et al. [2] used the linguistic features of the n-most-popular friends of a user on Twitter, related to homophily. Their classifier achieved an accuracy of 80.0% for binary gender classification.

Filippova [6] predicted the gender of YouTube users based on three types of feature: (1) character-based features, including average comment length, the ratio of the number of capital letters to the total number of letters, and the ratio of punctuation characters to the total number of characters; (2) token-based features, including the average comment length in words, the ratio of unique words to total tokens, lowercase unigrams with a total count over all comments (the 10,000 most frequent unigrams were used, the frequencies were computed based on a separate comment set), and the use of pronouns, determiners, and function words; and (3) sentence-based features, including the average comment length in sentences and the average sentence length in words. Their results showed that a user’s gender can be predicted from their social environment with an accuracy above 90% and from language alone with an accuracy of 89%. Bamman et al. [4] presented a study of the relationships between gender, linguistic style, and social networks using a novel corpus of 14,000 Twitter users and nine million tweets. A range of linguistic styles and topical interests were identified, which were clustered into male and female groups. Flekova et al. [7] employed surface, syntactic, punctuation, readability, semantic, and lexical features from online blog posts to build models that classified age and gender with an accuracy of 53% and 58%, respectively. These models were extended by adding the relationships between stylistic and syntactic features and the authors’ age and income, which were extracted from social media data. They found that most individual surface features correlate with age more strongly than with income, with the exception of punctuation and words longer than five characters [31].

### Research on instagram

Instagram is one of the most popular social networking sites and has high levels of user engagement. According to a recent Pew Research report, 35% of all U.S. adults use Instagram, and 60% of Instagram members used the platform daily in 2018 [32]. Because of its popularity, Instagram has been the subject of a substantial number of studies, including exploring the relationship between image content and engagement [33], analyzing image content and user types [34], studying tag-based “Like networks” created by users who share common tags [35, 36], and creating selfie categories using tags and clustering [37].

## Research goals

Our literature review indicates that many studies have identified and modeled user attributes on social media. The main content type analyzed in prior research has been text, with most studies focusing on complete sentences (e.g., from blogs) or a reasonable length of text (e.g., 140 characters from tweets). In previous Instagram studies, Jang et al. [35] focused on posting activity, Hu et al. [34] on image categories, and Deeb-Swihart et al. [37] on selfie categories using tags and clustering. However, images and tags have drawn relatively less research attention in the study of social media, and little is known about the extent to which either feature, or a combination of the two, can aid in characterizing users, or how images and tags are correlated with user characteristics. The goal of our study is thus to examine how images and tags characterize users and to build prediction models that classify users based on their attributes and that are broadly comparable to those models based on NLP features.

## Study design

**Fig 1:**
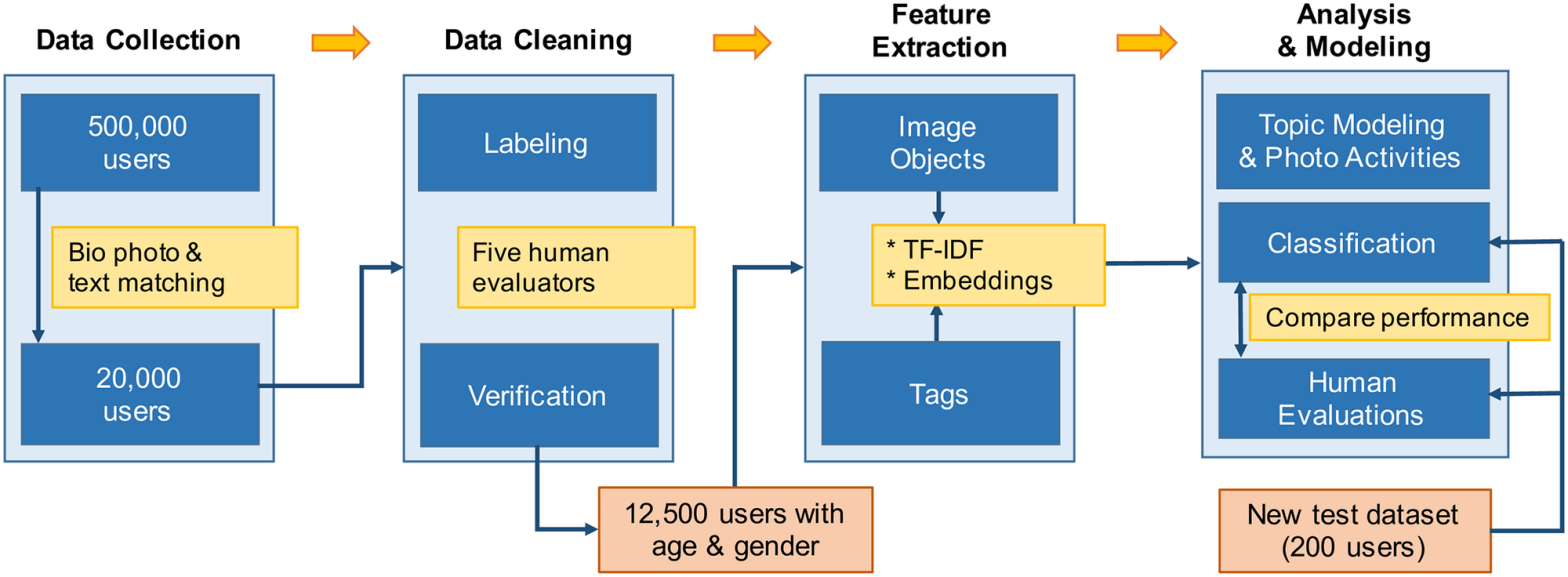
The study process used in the present work. We compared images and tags under the same conditions using TF-IDF and word embedding (Word2Vec).

Our study consisted of four distinct steps: data collection, data cleaning, feature extraction, and analysis and modeling (Fig 1). During the analysis and modeling step, we conducted a performance comparison between our models and human evaluations. The following sections detail each step.

### Data collection

Data collection was conducted in March 2017. We first chose 10 random seed users and crawled followers of these users until we reached 10,000 users. We then randomly chose 1,000 users (10%) from the pool of 10,000 and again crawled their followers until we reached 500,000 users. We used this two-step random-seed crawling process to minimize the bias in sampling a homogenous population. We complied with the terms of service for Instagram.

After the initial data collection, we defined our target user populations. We employed Erikson’s eight stages of psycho-social development [38]. We chose two age groups, adolescence (ages 13-19) and early adulthood (ages 20-39) because they are the primary user groups on Instagram [20, 21]. For gender, we considered males and females.

After defining the user groups, we began sorting our users into these groups. Classifying users into a specific age group was challenging because most social media platforms, including Instagram, neither collect nor disclose users’ age information. Here, we exploited two existing sources of information—the user’s profile photo and bio description—using face detection and text matching techniques. For face detection, we used Face++ (https://www.faceplusplus.com/), which is specifically designed for facial recognition. For text matching, we used a set of keywords that indicate age and gender information, such as “I am 30 years old” and “I am 15” for age, and “male,” “female,” and “proud mom/dad” for gender. As a result, we were able to find 20,000 users with age and gender labels.

When determining age using face detection, although Face++ generates relatively reliable results, there were many inaccuracies for several reasons (e.g., brightness, blur, color, user’s face not completely within the photo frame). Because the average standard deviation of the Face++ results for our sample was 5.1 years, and because of the difficulty of distinguishing similar ages (e.g., 19 vs 20), we added a 10-year gap between the two age groups to avoid any possible overlap between them. For simplicity, we refer to these final age brackets as *teens* (ages 13-19) and *adults* (ages 30-39). For gender, Face++ was generally reliable, with an accuracy of over 90% according to manual verification.

### Data labeling and verification

Obtaining accurate data prior to analysis is a critical step. To accomplish this, we recruited five participants to manually classify all users by gender and age. To facilitate this process, we built a simple website that displayed images and the profile information for each user. We masked account names and any links to social networking sites to minimize potential privacy issues. During this evaluation stage, we only considered classifications on which at least four of the five participants agreed. For users who did not meet our criteria, we asked the participants to evaluate those users again until at least four agreed. The entire labeling process took approximately two weeks. Our sample consisted of real people (e.g., no bots or organizations) and did not contain spam or pornographic content.

### Image object extraction

**Fig 2:**
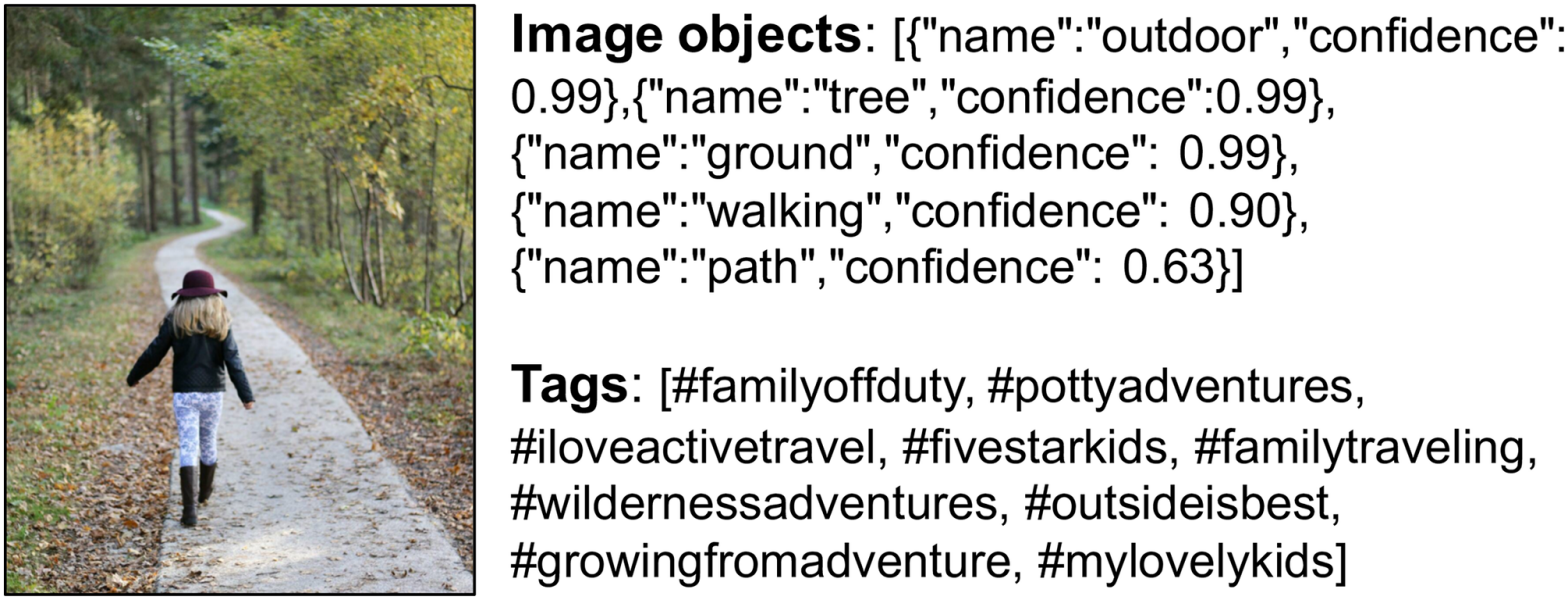
Example of an image with image objects (name and confidence) from an image recognition API and with user-generated tags. Mean±SD of image objects and of tags per image is 5.6±9.4 and 7.0±19.5, respectively.

To retrieve image information, we used Microsoft Azure Cognitive Services (https://azure.microsoft.com/en-us/services/cognitive-services/face/). This service allows researchers and developers to extract the content of an image by analyzing it with machine-learning models. It classifies images into thousands of categories, detects individual objects and faces within the images, and finds and reads printed words contained within the images. It offers a number of detection features, such as label, face, landmark, and logo detection. We used the label and face detection features for our analysis. The accuracy of each detection is included in the results. During the data processing stage, we used image objects with accuracies greater than 70%, 80%, and 90% for different test scenarios. Overall, Table 1 summarizes the data used in this study, including the number of users, images posted, image objects extracted from the service, and user-generated tags. Fig 2 illustrates an example of an image with its extracted image objects and user-generated tags.

**Table 1:** Summary of the data used in this study.

*Type*	*# Users*	*# Images*	*# Objects*	*# Tags*
Teen	5,111	45,133	342,148	340,074
Adult	6,968	103,931	504,309	700,590
Male	4,088	47,516	282,152	387,661
Female	7,991	98,548	564,305	653,003

## Results

For topic modeling and classification, we used the scikit-learn [39] and gensim [40] libraries implemented in Python. We used analysis of variance (ANOVA) as the statistical analysis.

### Topic modeling

We examined how the image objects and tags that people share could be identified and classified. To accomplish this, we created separate vector spaces for image objects and tags and used latent Dirichlet allocation (LDA) [41] to extract topics from the vector spaces. We initially set the number of topics to 50 and clustered the 50 topic results into 10-15 main topics. Because manual topic labeling was needed [42], we repeated this process for each group (i.e., males, females, teens, and adults) until we reached the same set of topics.

**Fig 3:**
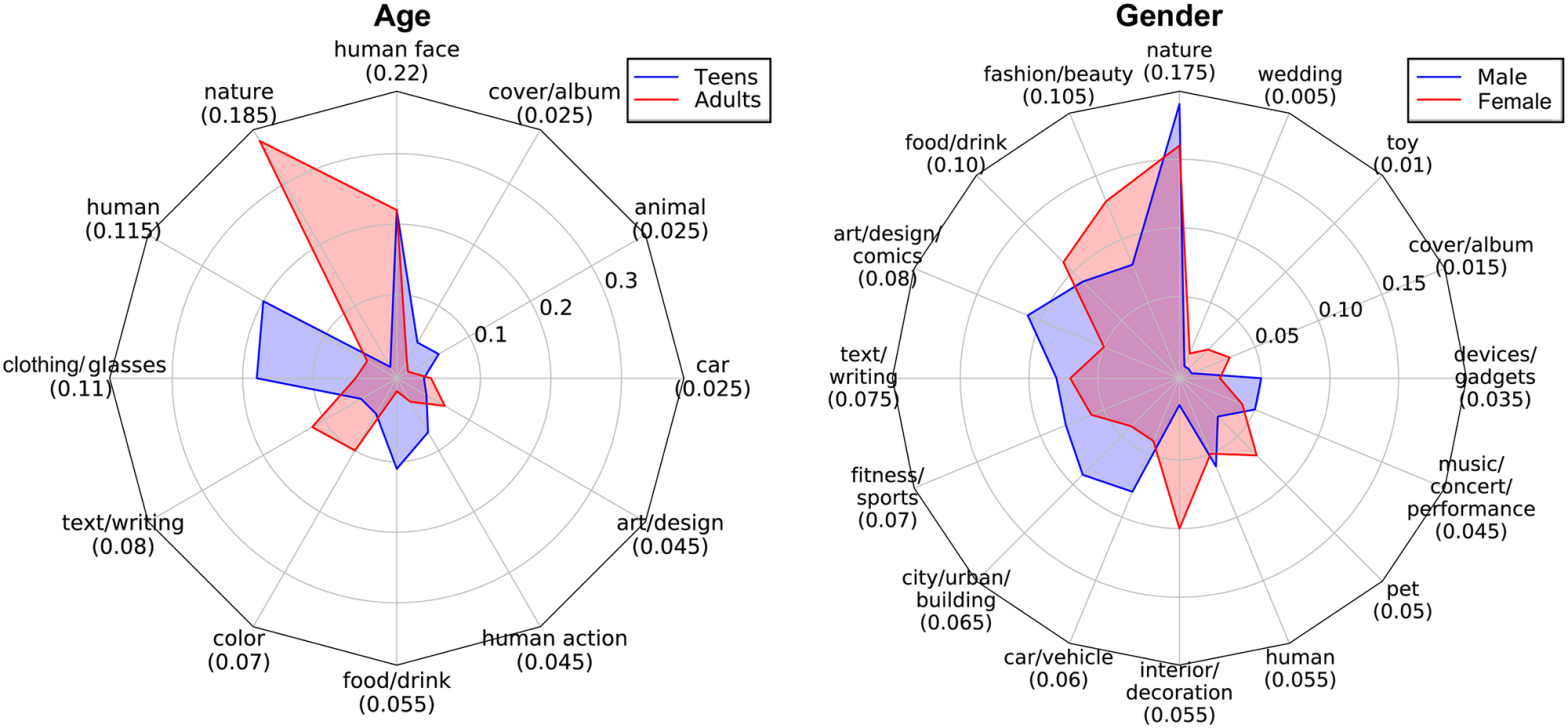
Differences in ratios between the age and the gender groups based on image object-based topic modeling. The number below each topic name is its ratio to all topics for all users. For example, 18.5% of the image objects are related to nature for both age groups combined. However, 37.5% of adult topics are related to nature (red), compared to 0.0% of teen topics.

**Fig 4:**
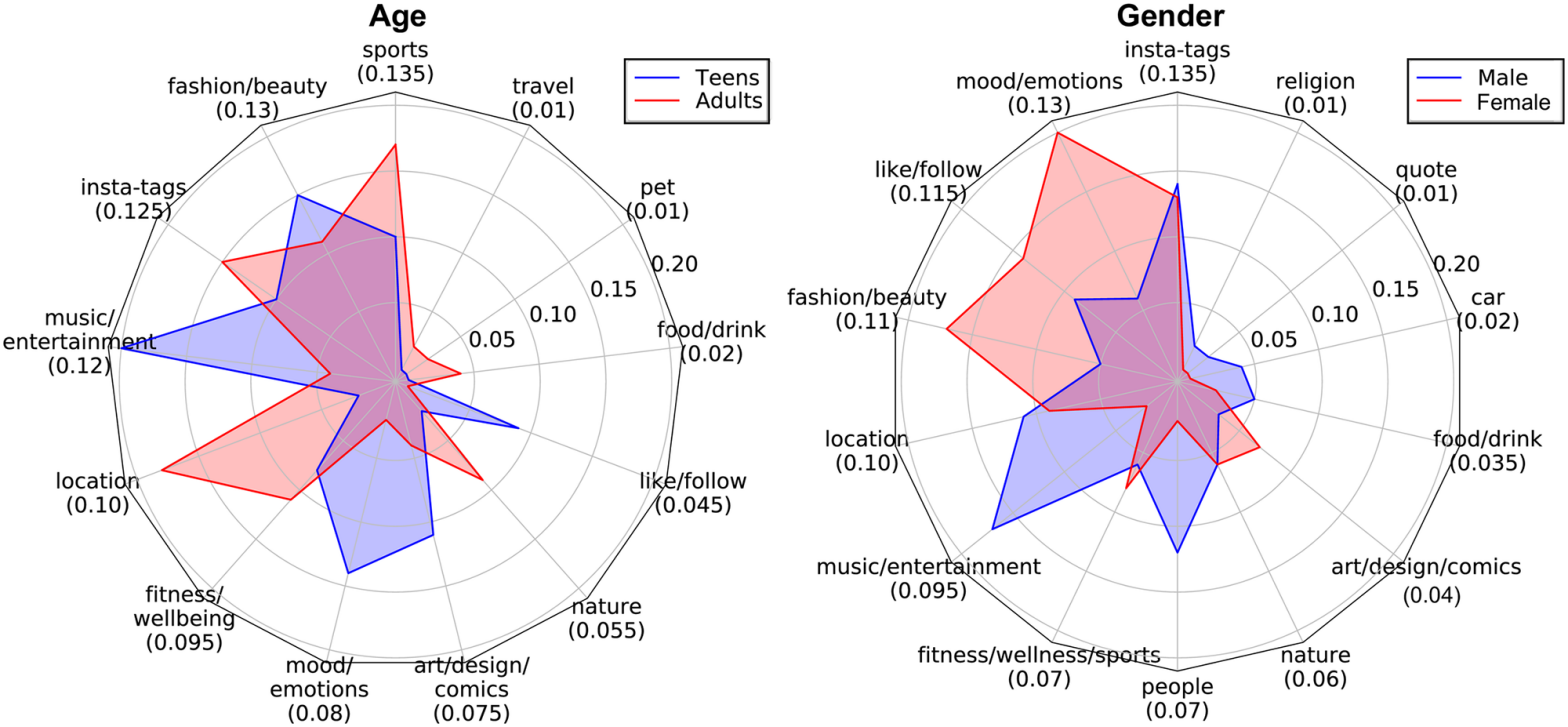
Differences in ratios between the age and the gender groups based on tag-based topic modeling. The number below each topic name is its ratio to all topics for all users. Compared to image-based topics, tag-based topics show distributions with higher variance.

Figs 3 and 4 summarize the difference in the frequency of the topics for the two age and gender groups based on images and tags, respectively. For the image object-based topics (Fig 3), several interesting patterns stand out. First, the topics identified based on age and gender were notably different. For example, age was clearly characterized by human-related topics (e.g., humans, human faces, and human actions), which was not the case for gender. Additionally, topical differences between the gender groups were more diverse than those between the age groups. Secondly, topics that were more closely related to humans (i.e., clothing/glasses, human actions, humans, and human faces) were significantly more common in the teen group than in the adult group. This indicates that many of the images uploaded by teens include humans. Third, the most common topic in the adult group was nature (e.g., mountain, sky, ocean, sun). However, nature did not appear as a major topic for teens. This indicates that adults are more likely to share images of their surroundings. Fourth, the topics of interest for females were more diverse than those for males. It appears that males had a greater focus on art/design/comics, cars/vehicles, and cities/urban areas/buildings, while females had a greater focus on fashion/beauty and interior/decoration.

Clear differences between the age and gender groups were also observed in the tag-based topics (Fig 4). First, topics including like or follow (e.g., #likeforlike, #followback, #followme), mood/emotion (e.g., #awesome, #happy, #sad, #tired), and music/entertainment were more frequently found accompanying teen images than adult images. Tags that fall under the like/follow topic represent the desire for the posted image to receive more likes and for the user to gain more followers. Tags in the mood/emotion topic represent teens’ tendency to express their emotional status through their images. Similar to the results in Fig 3, nature was found to be a topic that represents adults. Second, the mood/emotion and fashion/beauty topics were more heavily represented in females’ tags, while cars, music/entertainment, and people were more common in males’ tags.

### Selfies

During our analysis, we observed that many users in our sample uploaded images including themselves, their friends, or family. One of the primary functions of social networking sites is to distribute personal content that includes the faces of people. This creates and maintains social interactions and engagement between users [43]. The volume of selfies uploaded to social media each year is significant. For example, Google reported that 24 billion selfies were uploaded in 2015 (http://www.dailymail.co.uk/sciencetech/article-3619679/What-vain-bunch-really-24-billion-selfies-uploaded-Google-year.html). Because we observed clear differences between the age and gender groups, we subsequently analyzed the use of selfies to further examine the differences in posting behavior between our target user groups. To identify a selfie, we searched for images with hashtags such as #selfie, #myself, or #me.

**Fig 5:**
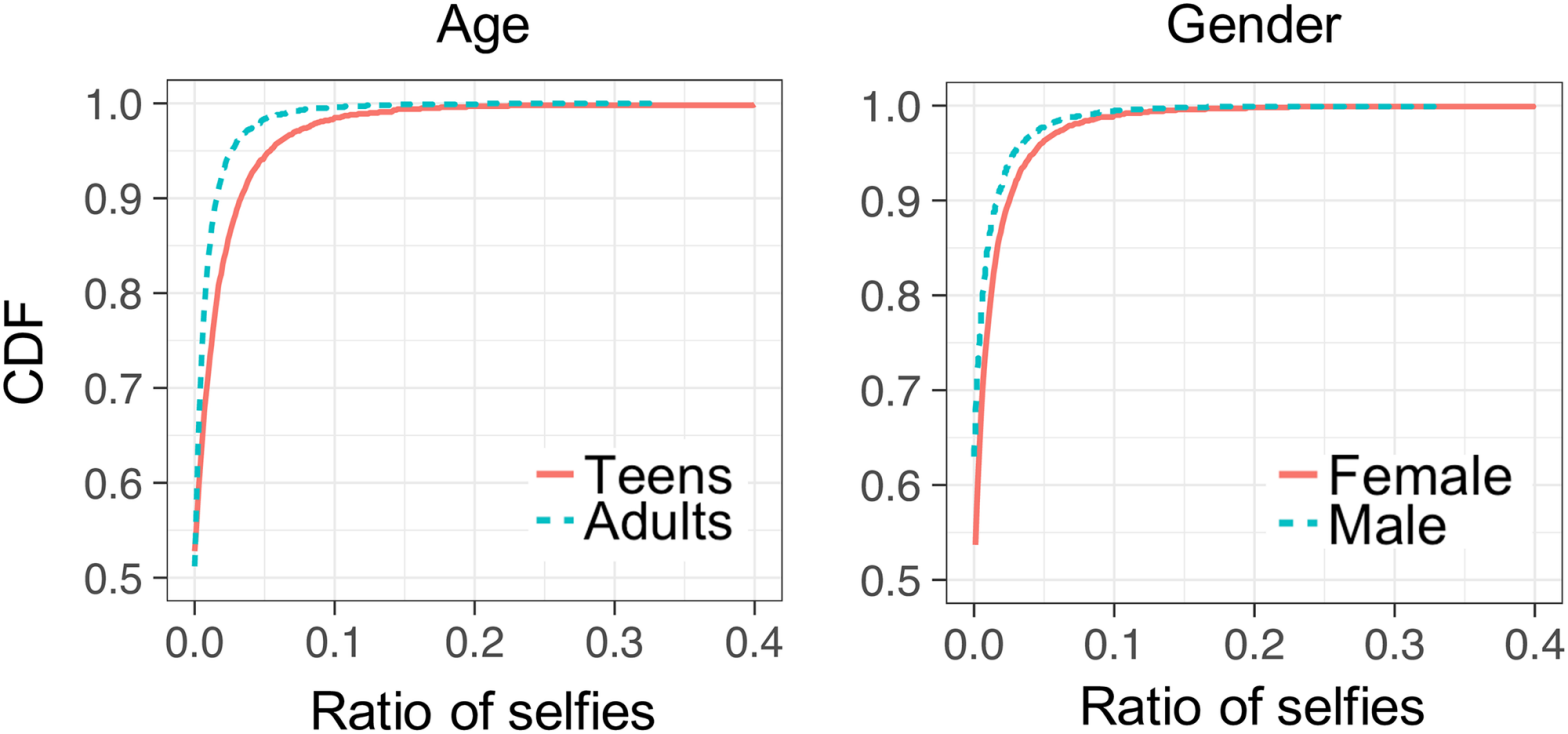
CDF of the ratio of selfies per image.

Fig 5 illustrates the cumulative distribution function (CDF) of the ratio of selfies to all images for each user group. Teens were more likely to post selfies than adults, which is somewhat in agreement with the findings from the topic modeling, in which it was observed that teens post more images containing topics related to the properties of humans. In terms of gender, although the difference was smaller than for the age groups, females were more likely to post selfies than males. Prior studies have also found that females tend to post more individual or partner selfies than males do. One of the main reasons is that females tend to exhibit higher social exhibitionism and extraversion than do males [44, 45].

### User classification

Our classification goal was to determine whether we can build classification models based only on image objects and/or tags (without using other features of the text, user, or network). We trained age-group classification models using a dataset consisting of vectors for image objects and tags. To create the vector space, we used term frequency-inverse document frequency (TF-IDF) [22] and word embedding (Word2Vec with bag-of-words implementation) [23, 24] with various feature sizes (100, 500, and 1000). The models with 500 features generated the optimal performance and were thus used for further analyses. We used TF-IDF to determine the importance of the image objects and tags. We used Word2Vec to determine the meaning and relationships of the image objects and tags.

We used logistic regression (LR) and random forest (RF) as our machine-learning models, which are widely used and which have exhibited good overall performance. We used 5-fold cross-validation (i.e., 80% for training and 20% for testing, repeated 5 times) to predict age and gender. We trained and tested the models for accuracy thresholds of 70%, 80%, and 90% for image object detection.

Tables 2 and 3 summarize the performance of the models. Overall, the models performed better for age detection than for gender detection regardless of the type of data (image objects or tags) or feature (TF-IDF or Word2Vec) at *p* < 0.05. It was also found that the models performed better with image objects than with tags (*p* < 0.05), indicating that images provide information that is more suitable for classification.

**Table 2:** F1 score of age classification (feature size of TF-IDF and Word2Vec is all 500; TF-IDF + Word2Vec is 1,000). In general, models from image objects showed greater performance than those from tags. For combined features, we used TF-IDF and Word2Vec, and the model performance increased when the combined features were used. 0.88 is the highest performance.

Age	Image Objs		Tags		Combined	
*Features*	*LR*	*RF*	*LR*	*RF*	*LR*	*RF*
TF-IDF	0.87	**0.88**	0.85	0.86	0.86	0.87
Word2Vec	0.84	0.85	0.71	0.78	0.78	0.81
TF-IDF + Word2Vec	0.84	0.86	0.84	0.86	0.82	0.84

**Table 3:** F1 scores of gender classification (TF-IDF and Word2Vec feature size = 500 each). Compared with Table 2, identifying gender was more difficult than identifying age. Model with image objects yielded greater performance than those with tags. For combined features, we used TF-IDF and Word2Vec, yielding an F1 score of 0.74.

Gender	Image Objs		Tags		Combined	
*Features*	*LR*	*RF*	*LR*	*RF*	*LR*	*RF*
TF-IDF	0.71	0.68	0.58	0.57	0.61	0.61
Word2Vec	0.63	0.62	0.56	0.49	0.61	0.58
TF-IDF + Word2Vec	**0.74**	0.64	0.63	0.49	0.68	0.58

The models also yielded greater performance with TF-IDF than word embedding, suggesting word importance is more influential than word meanings or relationships for modeling. When both features were used, the model performance increased in some cases. In particular, the higher performance (0.74 in Table 3) for gender detection is noticeable. Finally, the models did not exhibit any improvement in performance when both image objects and tags were used together. When combined, it appears that model performance was mainly influenced by the features from the image objects.

#### Feature importance

**Fig 6:**
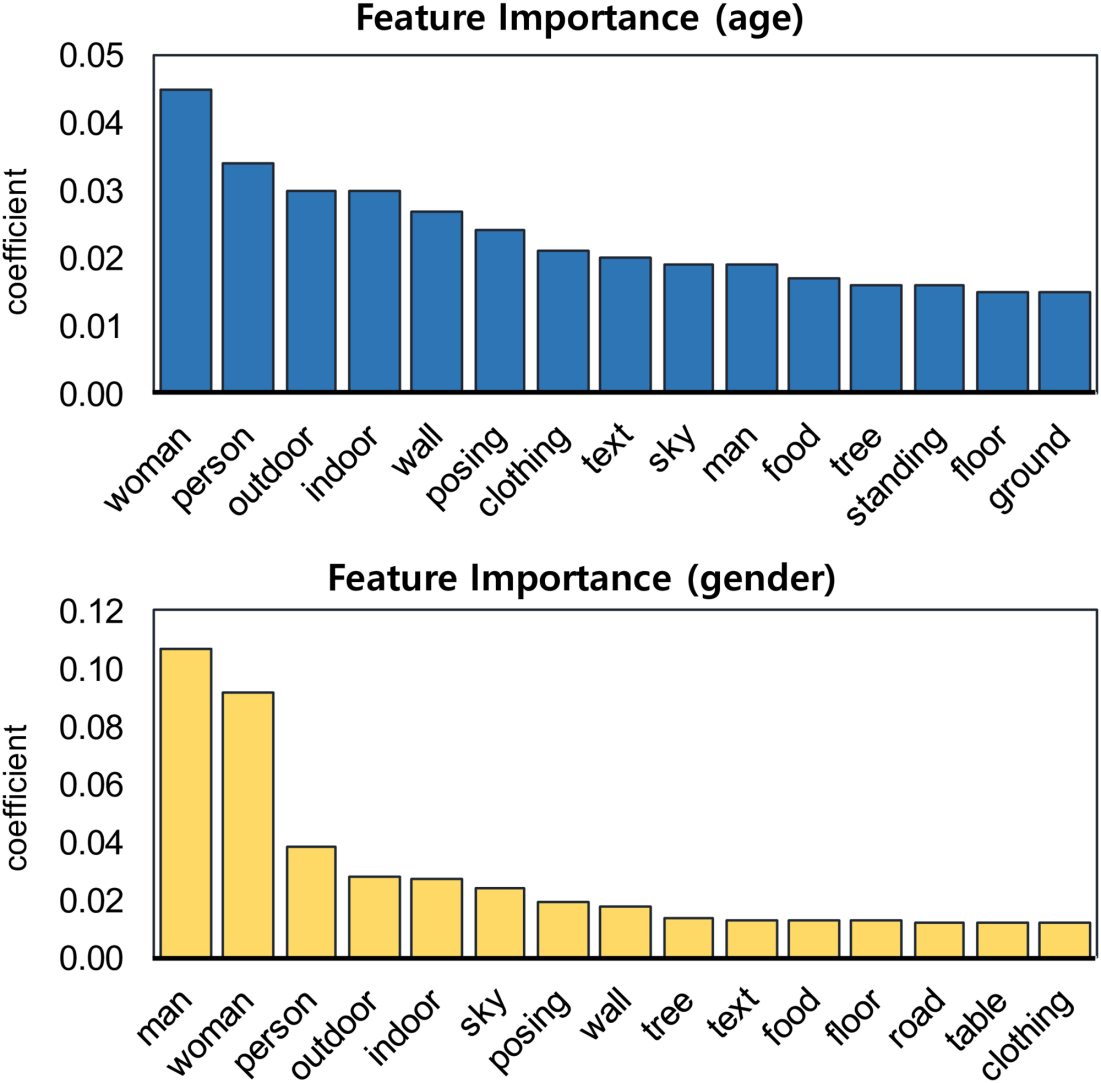
Feature importance for the random forest model based on image objects. The bars represent the feature importance of the forest, along with its inter-tree variability.

**Fig 7:**
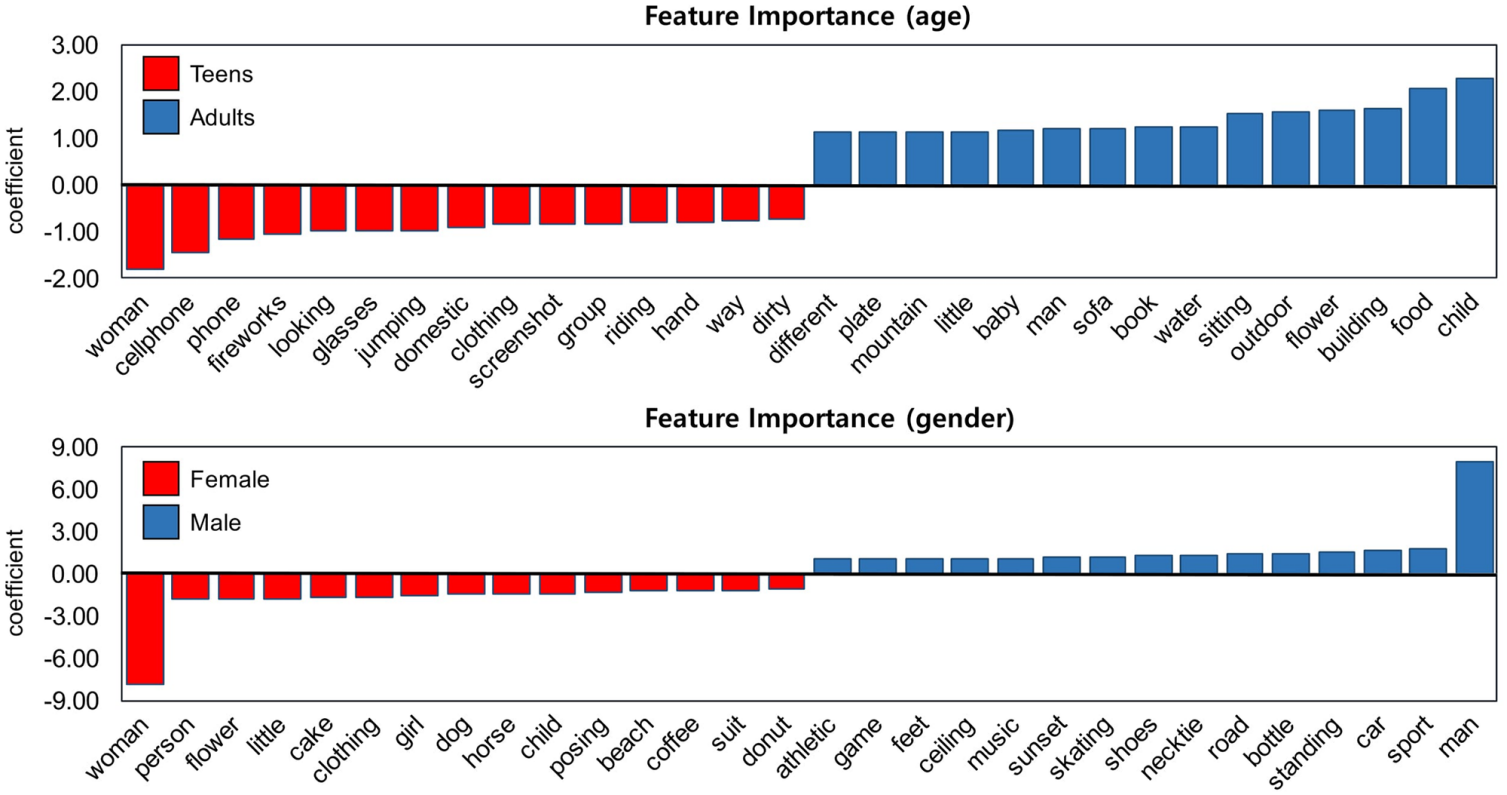
Feature importance for the logistic regression model based on image objects. For gender, only two words (woman and man) strongly affect the model, though the other words still have relatively high coefficients.

Based on the classification results, we further analyzed feature importance (i.e., the important objects). Because the logistic regression and random forest algorithms provide different perspectives on feature importance, we employed both in the analysis. Additionally, because the model that used image object data achieved the highest performance, we used that model to assess the feature importance. Fig 6 (random forest) and Fig 7 (logistic regression) present the top 15 most important features based on coefficients from the random forest and logistic regression models, respectively. These features were the top 15 average features from the models generated from 5-fold cross-validation. Overall, the analysis of feature importance demonstrated clear differences in image content between the age and gender groups. In the random forest model (Fig 6), human-associated objects (e.g., woman, person, posing, clothing, and man), surroundings (e.g. outdoor, indoor, and wall), and nature (e.g., sky, tree, and ground) had a strong influence on the classification of age. For gender classification, two specific words were the most influential—man and woman. When we compare these results with the F1 scores in Table 3, the lower performance for gender classification compared with age may be the result of having only a few strong features.

For the logistic regression model (Fig 7), the feature importance for each user group can be directly compared based on the coefficients. For age, the strongest features for teens included fashion and beauty (e.g., looking, glasses, clothing, and hand) and devices (e.g., cellphone and phone), while those for adults included nature (e.g., mountain, water, and flower), babies (many adults posted images of their children, which were described with little, baby, and child), and food (e.g., plate and food). For gender, similar to the results in Fig 6, woman and man were the most strongly influential words for characterizing the users’ gender. Although the other features seem less influential, their coefficients were still comparable to those for age. Features associated with food (e.g., cake, coffee, and donut), fashion (e.g., clothing, posing, and suit), and animals (e.g., dog and horse) were observed in the female group, while those associated with sports (e.g., athletic, game, skating, road, car, and sport) were observed in the male group.

### Classification performance by humans compared to machine-learning models

Although there have been many studies that have built prediction models to identify gender and age, our models are unique in that we only used TF-IDF and word embedding for image objects and tags. To further evaluate our models, we compared their performance with that of human evaluators. This also allowed us to determine why humans make mistakes or have less confidence in their decisions.

#### Preparing new test data

We noticed that many users create an online account name that incorporates their year of birth (e.g., chris1998, loveyourlife85). After data collection, we manually determined if users were teen or adult, or male or female, by visiting their Instagram page and examining the images they posted. Most (over 95%) of the age and gender information for the test users matched our expectations, with only a few corrections being necessary. Additionally, we confirmed that the users were not included as part of our previous data presented in Table 1. As a result, we collected 200 new users (consisting of 100 teens and 100 adults, 100 males and 100 females) to use as test data. We also collected all of the images and tags associated with these users.

The data for each user was organized in five different ways: (1) image objects only, (2) tags only, (3) image objects and tags together, (4) real images only (i.e., without image objects), and (5) real images and tags together. (The image objects were represented as text in this stage of the study.) For each of these cases, we compared the performance of our models with that of human evaluators, with our models utilizing image objects, tags, and image objects and tags together. This approach also allowed us to use human evaluations as a baseline for the performance of our models.

#### User study design (survey)

**Fig 8:**
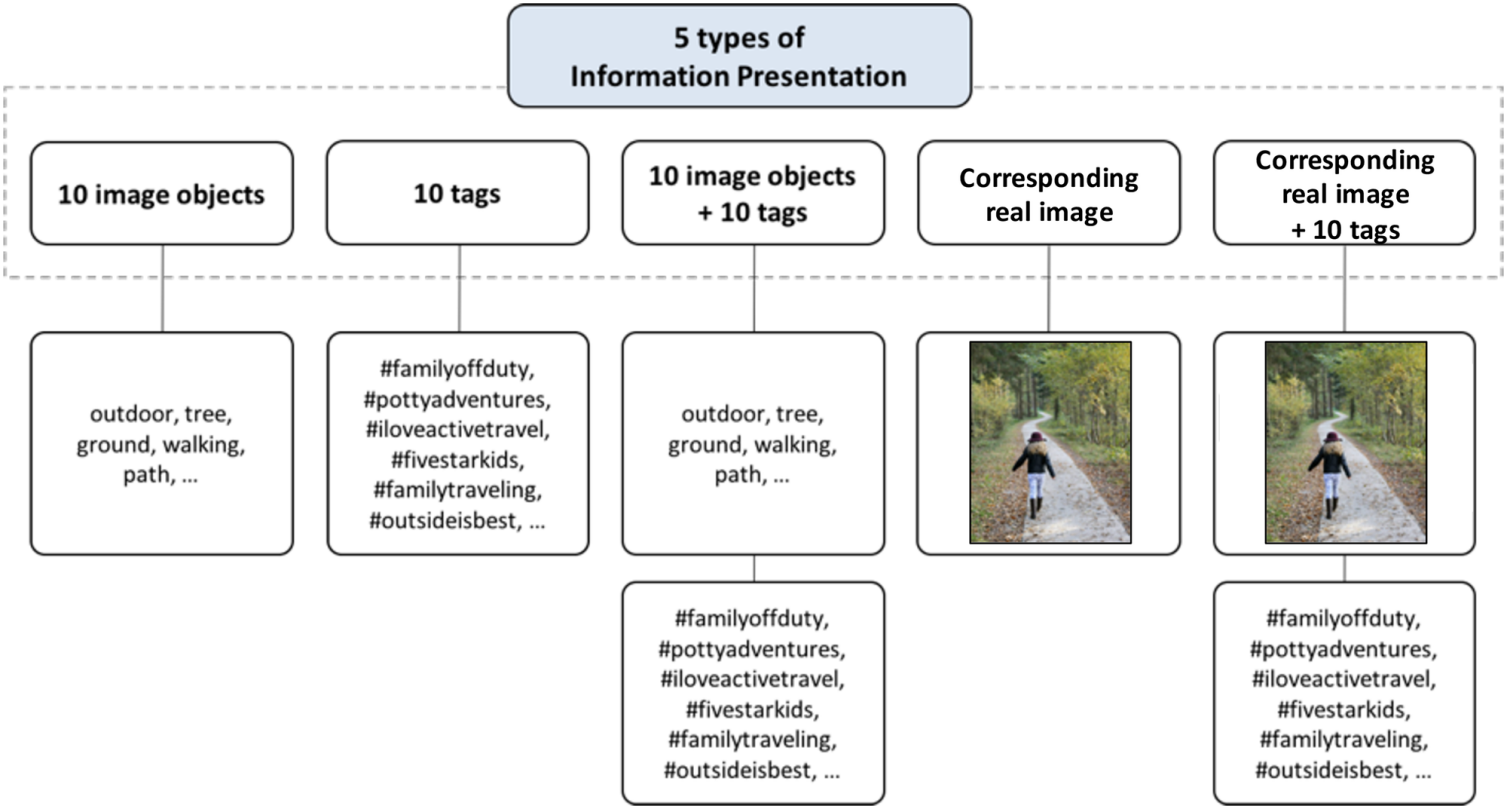
An example of the five types of information presented to the human evaluators. A single survey presented one of these information types for 50 different users (i.e., 10 users per information presentation type). Participants were asked to determine the age and gender of the user based on the presented information.

Our survey consisted of three main phases. First, we introduced our study and detailed the process for the participants who were to act as evaluators. Second, we asked the participants to indicate their age, gender, and length and frequency of their Instagram use. Third, the participants were asked to predict the gender and age of a given user and to report their level of confidence (based on a 5-point Likert scale, where 1 = not confident at all and 5 = very confident) in their prediction for five types of information: (1) 10 image objects written as text only, (2) 10 tags only, (3) 10 image objects and 10 tags together, (4) the corresponding real image from which the 10 image objects and 10 tags used in (1), (2), (3) were taken), and (5) the corresponding real image and 10 tags together (see Fig 8). Other than the image objects and tags, no additional identifiable user information was presented in the survey.

Based on the 200 test users, we created four surveys of 50 users each, with the users assigned randomly to each survey. Each of these surveys had five versions based on the different types of information described above, giving a total of 20 different surveys. Because many studies have demonstrated the reliability and validity of Amazon Mechanical Turk [46], we used this service to collect responses. To ensure the quality of responses, we included a filtering mechanism that allowed us to recruit participants who had a completion rate of at least 95%. Our study was reviewed and approved by the internal Institutional Review Board (IRB). We obtained informed consent from the participants at the beginning of the survey.

For each survey, we recruited 20 participants, thus our 20 surveys were completed by 400 participants in total. Each participant evaluated the age and gender of 50 users, thus we collected 20,000 responses. Each survey took approximately 15 minutes to complete (around 4 users per minute) and the participants were paid US$2.00 for their time. All of the responses were complete. When analyzing the results of the surveys, we chose the most common answer for each test user and compared this to the ground truth in order to calculate the accuracy of human evaluation.

**Fig 9:**
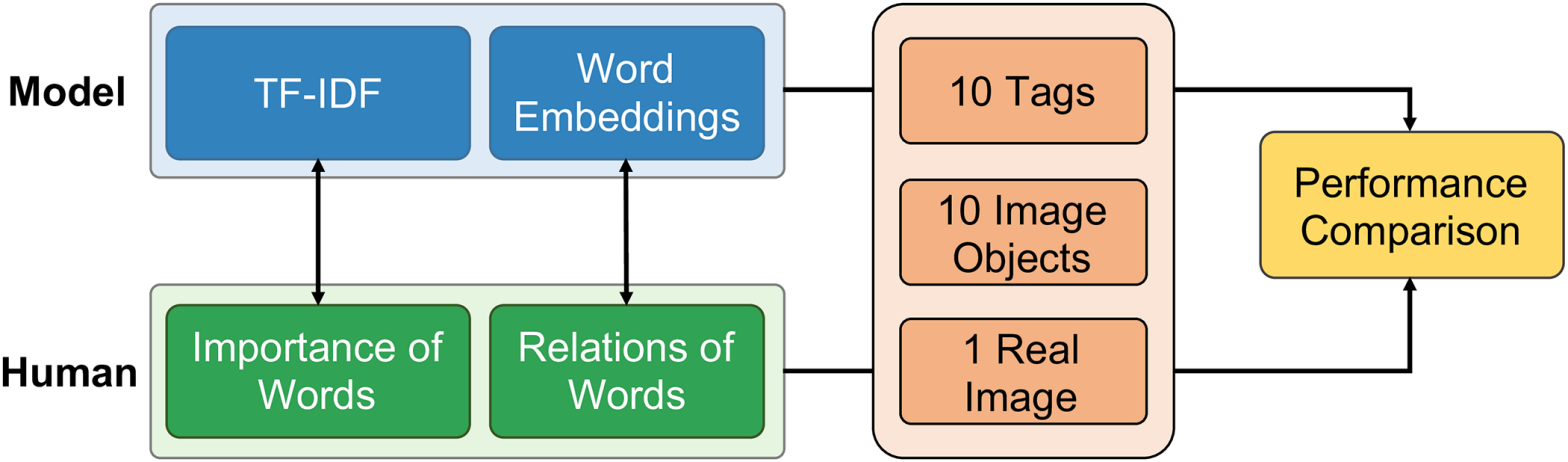
Schematic of the comparison of the proposed models and human evaluators under the same conditions. TF-IDF corresponds to the importance of words. Word embedding corresponds to the meaning and relationships of the words.

For model evaluation, we converted the image objects and tags of the 200 test users into the same vector space (TF-IDF and Word2Vec) as for our training data. We then ran our models using the converted vectors and obtained accuracy measurements for the new test sample with our original dataset. Finally, we compared the accuracy of our models with those of the human evaluators (Fig 9).

#### Performance comparison

**Fig 10:**
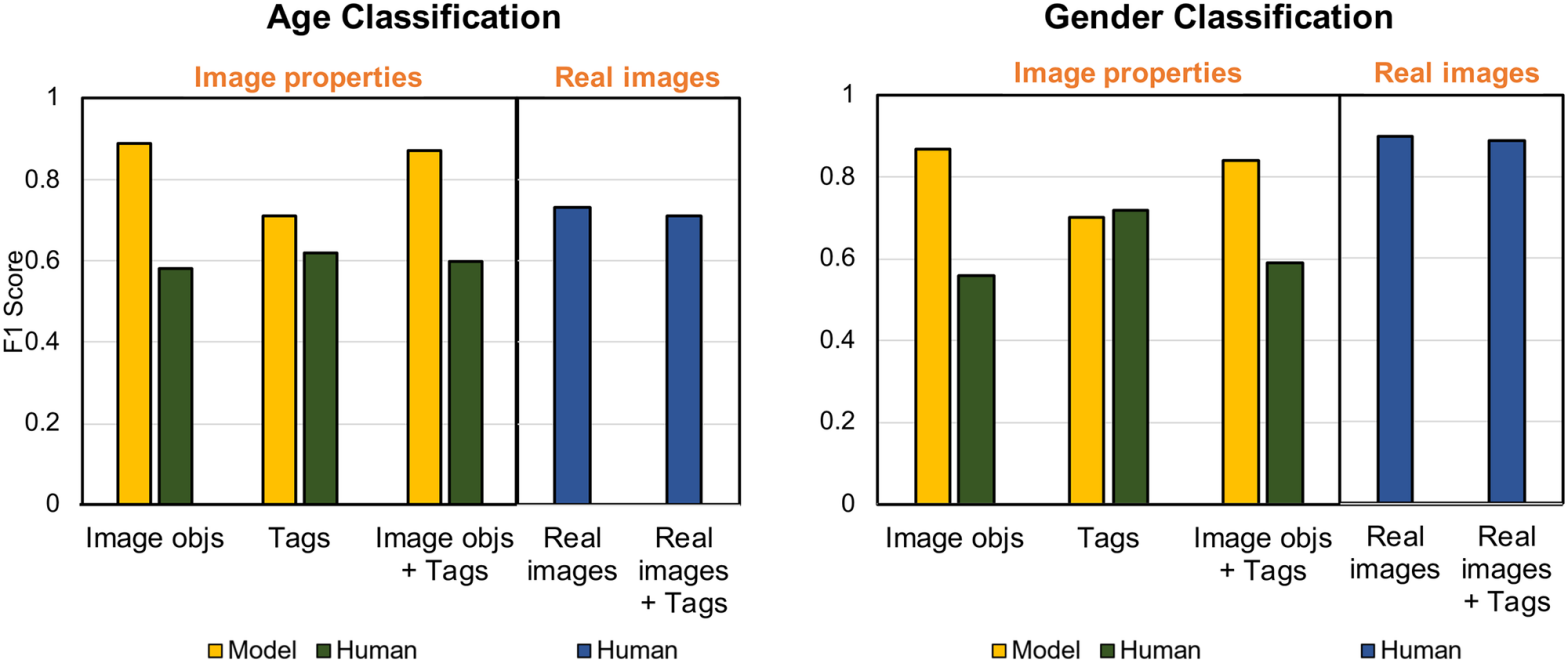
Performance results from 200 random images by the human evaluators. Two cases were considered: real images and real images + tags.

Fig 10 summarizes the age and gender identification performance of our models and the human evaluators for different types of information. We did not find any significant influence of the participants’ attributes (e.g., their age, gender, and length and frequency of Instagram use) on the accuracy and confidence of their answers.

When only image objects were provided, our models exhibited significantly higher performance than the human evaluators for age (0.89 vs 0.58 at *p* < 0.05) and gender (0.87 vs 0.56 at *p* < 0.05). This indicates that image objects appear to contain sufficient information for models to be able to accurately classify users, while human evaluators found these objects more difficult to interpret. This was reflected in the low level of confidence in the participants’ answers. They had an average confidence rating of 3.21±0.85 (mean±SD) for predicting age and 3.32±0.91 for predicting gender.

When only the tags were provided, our models displayed greater performance in detecting age than human evaluators (0.71 vs 0.62, respectively, at *p* < 0.05) but slightly lower performance in detecting gender (0.70 vs 0.72, respectively). Similar to the results from image objects, the levels of confidence for the tag data (age: 3.20±0.92, gender: 3.45±1.03) were low. More importantly, when we consider the models only, the model performance using tags was lower than that using image objects for both age (0.89 vs 0.71, respectively, at *p* < 0.05) and gender (0.87 vs 0.70, respectively, at *p* < 0.05). This indicates that image objects provide more distinctive information for age and gender classification.

When both image objects and tags were provided, classification performance did not change considerably when compared to that using image objects only for either the model or the human evaluators. However, the confidence levels of the participants’ answers did increase slightly (age: 3.51±0.88, gender: 3.61±0.96).

However, when real images were provided, significant differences appeared (Fig 10, blue bars). When we compared our models based on image objects with human evaluators using real images, our models still displayed significantly better performance for age group classification (0.89 vs 0.73, respectively, at *p* < 0.05). However, human evaluators exhibited slightly better performance than the models for gender classification (0.87 vs 0.90, respectively). Given that some images used in the study contained faces or were selfies (with which we initially expected that human evaluators would perform better), this result highlights the challenge of determining age for human evaluators even when faces are included in the image. The difference in accuracy in the age and gender classification by human evaluators was reflected in their level of confidence in their choices. They had a lower average confidence rating when predicting age (3.90±0.74) than when predicting gender (4.25±0.52).

Lastly, when both a real image and tags were provided, the performance of the human evaluators was slightly worse than that when using an image only (age: 0.73 vs 0.71, gender: 0.90 vs 0.89, respectively). It seems that including both types of information did not aid the human evaluators in making more accurate predictions. The confidence levels were similar to those with images only (age: 3.83±0.93, gender: 4.15±0.77).

In summary, these results demonstrate (1) the robustness of our models with a completely new set of test data, (2) the overall better performance by our models compared to human evaluators, and (3) the greater influence of images than tags in determining user characteristics. Additionally, the results for the human evaluators reflect the difficulty in assigning age and gender to a user, with many evaluators making incorrect decisions. This suggests that a meticulous study design is required when using human-generated annotations to label data because human classification can vary considerably depending on the data type and study conditions.

## Discussions

Data that represents human engagement on social media provides ample opportunities to understand how humans behave online. Based on this, we aimed not only to understand the behavioral patterns of user groups through a comparative analysis of large datasets of image objects and tags but also to create prediction models that classified users into teens or adults based on features derived from image objects and tags. We presented an in-depth comparative analysis of user image-sharing practices using deep learning techniques.

### Differences in posting behaviors

It was found that image objects and tags reflected different types of topic between the user groups. The differences in topic choices between teens and adults, and between males and females, were noticeable. Based on image objects, teens showed significantly more interest in topics related to humans (i.e., human actions, humans, and human faces). This could be related to selfie-posting behavior, which is also more frequent for females. This study found evidence of females using hashtags as a form of self-representation. This agrees with prior research, which has reported that the frequent posting of selfies by teenage girls is greatly influenced by idealized beauty standards and peer portrayals of beauty standards [47, 48]. Similarly, other studies have found that females tend to be more likely to use social networking sites to compare themselves to others and to search for information [49]. Nature was another major topic that distinguished teens and adults. When we examined tag-based topics, interest in nature and location was significantly higher among adults than among teens. This suggests that a high proportion of the images uploaded by adults include their surroundings (even when the images include humans), whereas most images posted by teens depict humans, faces, or actions.

Using tag-based information, females showed greater interest in fashion/beauty and interior/decoration, while males showed greater interest in cities/urban areas/buildings and devices/gadgets. This reflects common gender stereotypes and indicates that the gender differences inherent in many behaviors are mirrored in online spaces [49]. In terms of age, teens showed greater interest in likes/follows than adults. It is clear that teens attempt to gain followers and receive more likes by making their activities and images more visible to others, which reflects teen commenting behaviors in response to random users with whom they do not share a social connection [50, 51]. These motivations do not seem to be as pervasive for either images or tagging activities for adults.

### User classification

The behavioral characteristics of social media use, which are based on user attributes, allowed us to build a classification model that achieved good performance. In comparison to previous NLP-based studies, in which their models produced classification accuracies of between 75% and 91%, the highest performance achieved by any of our models was 88%. Given that our models were based only on the TF-IDF and word embedding of image objects and tags, while most prior models used a more varied range of features, we believe that our model produces an acceptable classification performance. It was also found that feature importance differed significantly between user groups. Because images are always available on image-based social media, user classification models that utilize features from images (e.g., objects) can be easily developed and applied.

### Study insights

For a given image, we had *O* image objects describing the image, and users themselves self-selected *T* tags that they felt were related to the image. Despite the acceptable performance of the deep learning model in this study, when the machine extracts *O* image objects, it can be assumed that there is some information loss. However, our study found that image objects were more useful for discriminating age and gender than tags. This raises an interesting question: Does *O* is higher than *T* in size on average, thus do tags provide more information? Interestingly, this was the opposite in our case, where *O* < *T*. The classifiers that employed images produced a higher classification accuracy than those using tags. The Mean±SD of *O* was 5.6±9.4 and of *T* was 7.0±19.5 per image. This means that, even with the smaller number, images better represented the user attributes analyzed in this study (age and gender) than did tags (*p* < 0.05).

The distribution of the topic modeling results also supports this. When we look at the results in Figs 3 and 4, the image-based topic modeling was more focused on fewer topics, while tag-based topic modeling exhibited a distribution with greater variance. For example, for the use of tags by gender, insta-tags and like/follow were used widely by both genders (3rd and 5th for males, 3rd and 4th for females, and 1st and 3rd for both groups combined, respectively). This reduced the performance of the models. We can therefore conclude that even though more tags were associated with the images, many of the tags had less influence on the accuracy of user classification than did the images.

Another interesting finding was that human raters were less accurate than the models in classifying users by age and gender. We first used human subjects to validate the ground truth labels for the training data for modeling, for which both the user’s profile and posted photos were available, and then used them to compare their classification performance to that of the machine-learning models for a new test dataset. In the second case, the human evaluators and the models were given only the users’ posted images, objects, and/or tags (i.e., no profile photo or bio) and asked to predict their age and gender. The lack of a profile photo or bio may have made the prediction task more difficult for the human raters and led them to perform worse than the models.

### Study implications

Many prior studies on social media have assumed that tags are representative of their corresponding content (including images) [18, 19]. This is indeed a valid assumption. However, our study demonstrates that image objects have significantly more influence than tags on the classification of age and gender. Thus, we believe the idiom *“a picture is worth a thousand words”* accurately applies to the classification of these particular user attributes on Instagram.

Social networking sites can use the insights from our study to identify user groups that have a strong similarity. The names of image objects and tags can also be used to match users with similar interests while also providing age and gender information. This approach could be used to create an interactive social space that extends beyond simply tracking followers and locations. In addition, because we found that images are more influential than tags in user attribute classification, social media platforms could create keyword suggestion tools via deep learning using images rather than relying on text-based information. We believe these functions can provide users with more specifically tailored suggestions of who to follow and what to see. It also provides users with the opportunity to discover and interact with new people who have similar interests and/or are similar in age or gender. They can also be applied to commercial services, such as product recommendations.

One concern for recommendation services is privacy. Therefore, privacy-preserving design decisions for implementing these features, such as allowing users to control their visibility, should be considered. However, from a different perspective, our models allow the possibility of combining users’ attributes and posting behaviors (e.g., images, tags, comments) to determine if a user displays abnormal or potentially risky posting behaviors or interactions. For example, studies have reported that 88% of social media-using teens have witnessed other users being cyberbullied on social networking sites [52], and 72% of adolescents had experienced at least one incident of cyber-bullying in the previous year [53]. Additionally, researchers have actively been investigating the characterization of users who may suffer from mental illness (e.g., depression) based on their social media posts, including images and text [54, 55]. Models based on our approach can detect users with certain user attributes (e.g., age and gender) and, using their behavior and activity data, identify unusual incidents, thereby enabling social networking sites to examine their behaviors and activities more closely and take appropriate action if required (e.g., the recommendation of images that may alleviate depression). As discussed earlier, one approach to handling potential privacy issues is to give full control to the user with respect to recommendations (some users may find recommendation results useful, some may not). Another strategy would be utilizing a user’s social network of friends. If an unusual incident is identified, the corresponding posts or activities can be shown to the user’s friends, allowing them the opportunity to act. Because the decisions are made and/or actions are taken by other users (close friends), data and behavioral manipulation could be controlled.

### Limitations and future work

Although our work yielded a number of interesting insights, there are some limitations that need to be addressed in the future. First, our results may not be generalizable because the original user sample used for classification directly provided their age information to some extent (either on their profile or through tags). In addition, users in their 20s were not considered, which limits the conclusions that can be drawn about age. The next step is to build more comprehensive classification models and to identify users and collect data that represent the diverse range of users on social media. Using information from a greater number of users is also necessary.

Second, a more comprehensive analysis can be achieved by combining the features from image and text processing (e.g., captions, tags, and comments) to extract more precise content from test images using additional image recognition services (e.g., Google Vision and Amazon Rekognition) that extract other features of images based on their own models. This analysis could include a study of temporal changes in image-sharing activities and the identification of image objects that potentially lead to privacy leaks, among other topics.

Third, because social media is an open space, there is a possibility that some of the users in this study lied about their age and/or gender in their bio. Similarly, there might be some level of discrepancy between the content that a user posts and that user’s real life or their intentions in posting that content. This is related to Erving Goffman’s work on self-presentation [56], which states that an individual’s online actions are subject to the mediated artifact of their performance being observed. While it would be challenging to identify these users, they may have some effect on future findings and insights.

Another important consideration for future research is to examine the number of features required for both machine-learning models and humans to produce an acceptable classification performance and how the number of features available affects that accuracy. This will allow us to examine whether accuracy improves more rapidly for models or humans and to determine the threshold at which adding an additional feature greatly improves accuracy. By doing so, we can understand how to better utilize the human perspective in building more useful and more accurate models.

## Conclusion

We demonstrated the strong influence of age and gender on the way people use Instagram based on an analysis of topical and contextual differences. Our models, which are based solely on the TF-IDF and word embedding of image objects and tags, yielded F1 scores of up to 88% for classifying age and 74% for classifying gender. We demonstrated the robustness of our models using a new set of test data and performing a comparison with human evaluators, proving that the adage *“a picture is worth a thousand words”* is valid in the classification of user attributes. Our study highlights that future research should exploit the information contained in images to a greater degree because it can be used to develop more accurate classification models, especially in the absence of accompanying text.
